# The role of nutrition rehabilitation in the recovery of survivors of critical illness: underrecognized and underappreciated

**DOI:** 10.1186/s13054-022-04143-5

**Published:** 2022-09-08

**Authors:** Lesley L. Moisey, Judith L. Merriweather, John W. Drover

**Affiliations:** 1 College of Pharmacy and Nutrition, University of Saskatchewan, E3126 Health Sciences Building, 104 Clinic Place, Saskatoon, SK S7N 2Z4 Canada; 2 Department of Critical Care, Royal Infirmary of Edinburgh, 51 Little France Crescent, Edinburgh, EH16 4SA UK; 3 Department of Critical Care Medicine, Queen’s University, Davies 2, 76 Stuart Street, Kingston, ON K7L 2V7 Canada

**Keywords:** Critical care, Intensive care, Nutrition assessment, Nutrition therapy, Energy intake, Protein intake, Care transitions

## Abstract

Many survivors of critical illness face significant physical and psychological disability following discharge from the intensive care unit (ICU). They are often malnourished, a condition associated with poor outcomes, and nutrition remains problematic particularly in the early phases of ICU recovery. Yet nutrition rehabilitation, the process of restoring or optimizing nutritional status following illness, is seldom prioritized, possibly because it is an underrecognized and underappreciated area in critical care rehabilitation and research. To date, 16 original studies have been published where one of the objectives includes measurement of indices relating to nutritional status (e.g., nutrition intake or factors impacting nutrition intake) in ICU survivors. The primary aim of this narrative review is to provide a comprehensive summary of key themes arising from these studies which form the basis of our current understanding of nutritional recovery and rehabilitation in ICU survivors. ICU survivors face a multitude of barriers in achieving optimal nutrition that are of physiological (e.g., poor appetite and early satiety), functional (e.g., dysphagia, reduced ability to feed independently), and psychological (e.g., low mood, body dysmorphia) origins. Organizational-related barriers such as inappropriate feeding times and meal interruptions frequently impact an ICU survivor’s ability to eat. Healthcare providers working on wards frequently lack knowledge of the specific needs of recovering critically ill patients which can negatively impact post-ICU nutrition care. Unsurprisingly, nutrition intake is largely inadequate following ICU discharge, with the largest deficits occurring in those who have had enteral nutrition prematurely discontinued and rely on an oral diet as their only source of nutrition. With consideration to themes arising from this review, pragmatic strategies to improve nutrition rehabilitation are explored and directions for future research in the field of post-ICU nutrition recovery and rehabilitation are discussed. Given the interplay between nutrition and physical and psychological health, it is imperative that enhancing the nutritional status of an ICU survivor is considered when developing multidisciplinary rehabilitation strategies. It must also be recognized that dietitians are experts in the field of nutrition and should be included in stakeholder meetings that aim to enhance ICU rehabilitation strategies and improve outcomes for survivors of critical illness.

## Background

Critically ill patients present with life-threatening conditions that require costly and sophisticated levels of care. Despite their high severity of illness, the global average intensive care unit (ICU) mortality rate is approximately 16% [1], which is in part due to advances in medical knowledge and technologies. Surviving an ICU stay marks the beginning of a long and arduous journey to recovery [2]. ICU survivors often face pronounced functional, cognitive and psychological impairment that impact both short- and long-term recovery [3, 4], the ability to return to work [5–8] and quality of life [5, 9, 10]. The term post-intensive care syndrome (PICS) has been coined to define this constellation of health-related morbidities and deficits experienced by survivors of critical illness that span across three broad domains encompassing physical, cognitive, and psychological functioning [11–13].

**Fig. 1:**
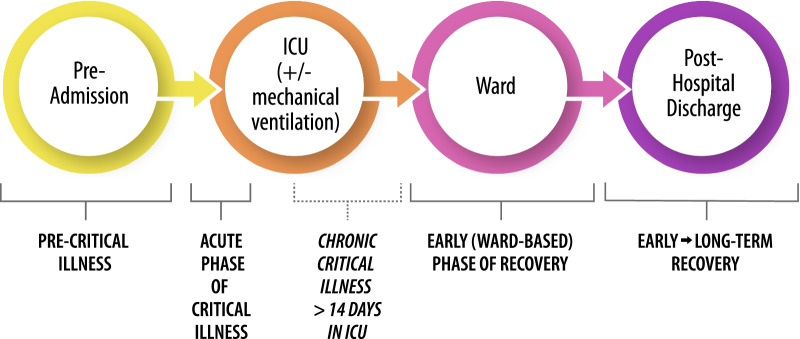
Significant phases along the trajectory of critical illness. Arrows represent important care transitions

Strategies and interventions to mitigate PICS, aid in physical and neuropsychological recovery, and enhance quality of life in survivors of critically ill patients are needed [3, 11]. Little attention has been given to the role of nutrition in the context of ICU recovery and rehabilitation, and representation from the nutrition field has been largely absent at multidisciplinary stakeholder and consensus meetings focused on improving ICU outcomes [3, 11, 14]. Since optimization of diet and nutritional status translates into improved function, cognition and mental health [15–18], nutrition should be considered an essential component to ICU rehabilitation and recovery. Several research gaps exist in this field of study, which contributes to an underappreciation of the role nutrition plays in facilitating recovery and improving health-related quality of life in ICU survivors. Thus, the aims of this review are to: (1) provide an overview of the theoretical basis underlying a supportive role for nutrition in the management or mitigation of PICS; (2) summarize and synthesize key findings from 16 foundational studies [19–34] that form the basis of our current understanding of nutritional recovery and rehabilitation in ICU survivors through the reporting of data on nutrition intake, barriers to intake and/or changes in nutritional status all within the post-ICU period; 3) discuss pragmatic strategies to enhance nutritional rehabilitation in ICU survivors in the early and later stages of recovery (Fig. 1) in the absence of formal practice guidelines; and 4) outline key research gaps in the field of nutritional rehabilitation for survivors of critical illness.

## The need for nutrition in post-ICU rehabilitation

### Critical illness is associated with malnutrition

**Fig. 2:**
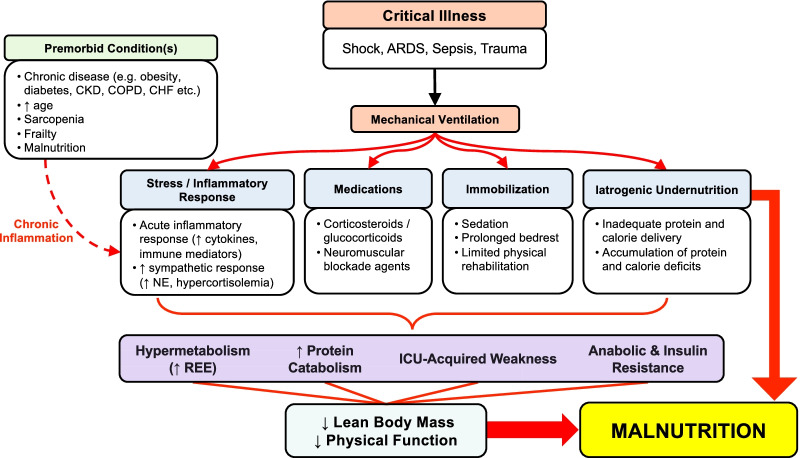
Factors influencing the development of disease-related malnutrition following the onset of critical illness. ARDS: acute respiratory distress syndrome; CHF: congestive heart failure; CKD: chronic kidney disease; COPD: chronic obstructive pulmonary disease; ICU: intensive care unit; and NE: norepinephrine

Contemporary definitions of malnutrition have evolved such that inflammation is recognized as a significant contributor to disease-related malnutrition [35, 36]. Patients admitted to ICU frequently have one or more premorbid chronic health conditions, and inflammation is present to varying degrees in both chronic [37, 38] and acute [39, 40] disease states. Inflammation is also associated with advanced aging [41, 42], and older adults, many of whom are frail and sarcopenic, comprise a large and increasing proportion of ICU admissions [43, 44]. The onset of acute illness triggers an acute inflammatory response and pronounced stress metabolism which results in increased catabolism, insulin resistance, and anabolic resistance [39]. Upregulated production of pro-inflammatory cytokines and mediators is associated with increased muscle catabolism resulting in a net loss of lean body mass, and reduced functional capacity and immune function, all of which are characteristics of malnutrition [45–47]. For a person to be diagnosed with malnutrition, they must exhibit one criteria of etiological origin (reduced food intake, reduced absorption or disease-related inflammation) and one of phenotypic origin (weight loss, low body mass index, or reduced muscle mass) [35]. Loss of muscle mass is a hallmark indicator of malnutrition and is a recognized symptom of PICS [48, 49]. In healthy individuals, skeletal muscle represents 30–45% of total body mass [50] and is important in regulating glucose disposal, protein turnover, immune function [51–53], and physical functioning [54]. Several factors are known to increase net muscle protein breakdown including inflammation, oxidative stress, immobilization, and corticosteroid use [39, 49, 55–57]. Critically ill patients also experience insulin resistance and anabolic resistance, which is a blunted anabolic response to amino acids characterized by failure to stimulate nutrient uptake and muscle protein synthesis and inhibit muscle protein breakdown [58, 59]. Muscle atrophy frequently occurs in the ICU with the steepest rate of loss occurring within the first  two weeks of ICU admission [60–64]. Furthermore, throughout the duration of mechanical ventilation, patients receive inadequate protein and energy and experience iatrogenic undernutrition [65]. Thus, due to the myriad of factors the critically ill experience, most patients requiring mechanical ventilation will be discharged from ICU with some degree of malnutrition (Fig. 2).

### Changes in body composition associated with critical illness

There is limited research evaluating longitudinal changes in body composition following critical illness. In a pivotal study by Herridge et al. [6] examining disability in ICU survivors, patients with acute respiratory distress syndrome lost 18% of their baseline body weight over the course of ICU admission, and one year following ICU discharge only 71% of surviving patients had returned to their preadmission weight. In a group of 136 ICU survivors, Kvåle et al. [66] reported 40% of patients lost greater than 10 kg, and 6 months following ICU discharge, 35% remained at their post-ICU weight and 15% had lost further weight. Weight represents a net sum of all tissues and cannot distinguish changes occurring in tissue (e.g., muscle and fat) compartments. While the return to pre-illness weight may be interpreted as a positive sign in recovery, four studies have reported that weight gain following critical illness is secondary to increases in fat rather than muscle mass [67–70]. This may have broader detrimental implications on functional and psychological recovery and emphasizes why nutrition, which influences body composition, should be considered in ICU recovery.

## Nutrition recovery and rehabilitation following ICU discharge

Nutrition rehabilitation refers to the process of restoring or optimizing nutritional status following illness. Our present understanding of the nutritional health of ICU survivors and factors influencing it in recovery is limited. The current foundation of knowledge centering on nutrition recovery and rehabilitation after critical illness is based upon observations from 16 studies [19–23, 25–34, 71]. The sample sizes used in the group of studies reviewed range from 8 to 193 (excluding one case study), all were authored or co-authored by dietitians, they originated from a variety of geographical locations representing North America, Europe, and Australia, and all have reported on varying indices relating to nutritional status (nutrition intake, factors impacting intake, body composition, physical function, and/or global assessment of nutritional status) using a variety of different methodologies (Table 1). Synthesis of the data reported in these studies revealed contextual factors driving malnutrition in post-ICU patients center on three predominant themes: (1) the biological effects of critical illness; (2) organizational and process factors; and (3) nutrition knowledge of health care providers.

**Table 1:** Key findings from studies examining indices of nutritional status following liberation from mechanical ventilation

Author, year published (country)	Study design	Primary objective(s)	Study population and initial sample size	Nutrition indices assessed and methodologies used	Key findings
Nematy et al., 2006 [19] (England)	Prospective, observational study	To investigate gut hormone concentrations in patients during ICU stay and following LMV and relate them to appetite and energy intake measures.	Critically ill adults requiring MV and anticipated ICU LOS > 3 days *n* = 16 ICU patients *n *= 36 healthy controls	**Energy intake:** Oral diet: Food records completed daily by nursing staff EN/PN: calculated from flow sheets in the medical record Healthy controls: 3-day food diary **Estimation of energy requirements to assess adequacy:** Compared energy intake of ICU patients to healthy control subjects **Factors affecting intake:** Appetite: VAS **Body composition:** ICU admission: weight, BMI, TSF, MAC ICU discharge: weight, BMI, TSF, MAC	In comparison with healthy controls, following ICU discharge, patients consumed ~ 52% of energy and had significantly lower appetite scores, higher nausea, and earlier satiety.
Peterson et al., 2010 [20] (USA)	Prospective, observational study	To assess protein and energy adequacy and identify barriers to oral intake in ICU patients prescribed an oral diet exclusively during the first 7 days following extubation.	Critically ill adults requiring MV for > 24 h *n* = 50	**Energy and protein intake:** Modified multiple-pass 24-h recall conducted daily study duration **Estimation of energy and protein requirements to assess adequacy:** BMI < 30 kg/m^2^: 25 kcal/kg admission weight, 1.2 g protein/kg admission weight BMI ≥ 30 kg/m^2^: 11 kcal/kg admission weight, 2 g protein/kg ideal weight (calculated using Hamwi equation) **Factors affecting intake:** Patients asked open-ended questions to identify barriers to intake **Body composition:** ICU admission: Weight, BMI, TSF, MAC **Global nutritional status assessment:** ICU admission: SGA	Over first 7 days post-extubation, mean calorie and protein intake never exceeded 55% (ranged from 34–55%) and 37% (ranged from 23–37%) of estimated requirements, respectively. Primary barriers to eating: poor appetite, nausea/vomiting, difficulty chewing/swallowing, and disliking the food.
Salisbury et al., 2010 [21] Click or tap here to enter text.(Scotland)	Pilot feasibility case study	To describe the role and issues raised around the implementation of using a GRA to deliver enhanced physiotherapy and nutrition rehabilitation for up to 7 weeks after critical illness.	Critically ill adult requiring MV for > 4 days (stroke, head injury and liver transplant patients excluded) *n* = 1	Measures taken weekly for 7 weeks following ICU discharge **Energy and protein intake:** Food record charts (completion of food records was part of the enhanced nutrition care delivered by the GRA) **Estimation of requirements to assess adequacy:** Schofield and Elia equations **Factors affecting intake:** Appetite VAS **Body composition:** Weight, MAMC **Functional status assessment:** Rivermead Mobility Index, Timed-Up-and-Go, 10-meter walk test, hand-grip strength	Over 7 weeks post-ICU discharge; calorie intake as a percent of requirements ranged from 70 to 215% and protein intake 66–258%. (note: patient received nutrition via NGT or PEG during this period and fed to promote weight gain); improvements in functional measures and appetite observed between weeks 0 and 7; weight not reported.
Salisbury et al., 2010 [22] (Scotland)	2 studies: 1) Service evaluation of care 2) Pilot feasibility RCT	1) Determine the ward-based physiotherapy and nutrition services patients currently received following ICU discharge. 2) Determine whether use of a rehabilitation assistant to provide enhanced physiotherapy and nutrition rehabilitation is feasible.	Critically ill adults requiring MV for > 4 days (stroke, head injury and liver transplant patients excluded) Study #2: Intervention group (assigned a rehabilitation assistant): *n* = 8 Control group (standard care): *n* = 8	Measures taken weekly and 3 months following ICU discharge **Energy and protein intake:** Food record charts (completion of food records was part of the enhanced nutrition care delivered by the rehabilitation assistant) **Estimation of requirements to assess adequacy:** Schofield and Elia equations **Factors affecting intake:** Appetite VAS **Body composition:** Weight, MAMC **Functional status assessment:** Rivermead Mobility Index, Timed-Up-and-Go, 10-meter walk test, hand-grip strength	Patients in the control and intervention group consumed a weekly median 102% and 115% of estimated calorie requirements, respectively, and 63% and 77% of estimated protein requirements. At 3 months post-discharge, patients in the control and intervention groups were consuming a median 70% and 113% of estimated calorie requirements, respectively, and 69% and 90% of estimated protein requirements.
Merriweather et al., 2014 [23] (Scotland)	Qualitative: Grounded theory	To examine organizational issues and barriers influencing nutrition care during the post-ICU hospital stay.	Critically ill adults requiring MV for > 48 h *n *= 17	Organizational factors influencing nutrition care were acquired through observation of usual care and semi-structured interviews	Three organizational factors influencing nutritional intake were identified: ward culture, system-centered delivery of care, disjointed discharge planning.
Walsh et al., 2015 [24] (Scotland)	Multicenter, randomized parallel group intervention trial	To determine the effect of increased physical activity and nutrition rehabilitation delivered during the post-ICU acute hospital stay via use of a rehabilitation assistant on mobility, quality of life and disability.	Critically ill adults requiring MV for > 48 h (TBI, intracerebral bleed, stroke, Guillain–Barre syndrome excluded) Intervention group: *n* = 120 Control group: *n* = 120	**Factors affecting intake:** ICU discharge, 3-, 6-, and 12-month follow-ups: Appetite VAS **Body composition:** 3-month follow-up: Weight, BMI **Global nutritional status assessment:** 3-month follow-up: SGA **Functional status assessment:** Weekly in hospital post-ICU discharge and 3-, 6-, and 12-month follow-up: Rivermead Mobility Index 3-month follow-up: Timed-Up-and-Go Weekly in hospital post-ICU discharge and 3-month follow-up: hand-grip strength	Nutrition intake data not reported. Patients in the intervention group reported higher satisfaction with eating and nutritional support.
Chapple et al., 2016 [25] (Australia)	Prospective observational study	To quantify the amount of energy and protein prescribed and delivered throughout hospitalization (ICU and ward) in critically ill patients with TBI.	Critically ill adults with TBI requiring ICU stay ≥ 48 h *n* = 37	**Energy and protein intake:** EN/PN: calculated daily from flow sheets in the medical record up to day 90 of hospitalization Oral diets: weighed food records 3 days per week (2 weekdays, 1 weekend day) up to day 90 of hospitalization **Estimation of requirements to assess adequacy and cumulative deficit:** Energy and protein prescriptions assessed by the hospital dietitians as part of standard care were extrapolated from the charts **Barriers to intake:** Interruptions to nutrient provision documented from patient medical records	On the ward, patients receiving EN exclusively received 89% and 76% of energy and protein requirements, respectively; patients on oral diets exclusively consumed 75% and 74% of energy and protein requirements, respectively. Cumulative energy deficits accrued daily were significantly greater for patients on oral diet vs EN (~ 800 vs 450 kcal/d); daily protein deficits, while high, were not significantly different between those on an oral diet vs EN (40 vs 37 g protein/d).
Merriweather et al., 2016 [26] (Scotland)	Qualitative: Grounded theory	To explore factors influencing nutrition rehabilitation during hospitalization following ICU discharge and at 3 months following ICU discharge.	Critically ill adults requiring > 48 h MV *n* = 17	Factors influencing nutrition care were acquired through researcher observation of usual care (1 h for 3 times weekly) and semi-structured interviews (weekly during patients stay on the ward and at 3 months post-ICU discharge)	“Inter-related system breakdowns during the nutritional recovery process” was the overarching category identified that influenced eating post-ICU. Major themes identified that influence nutritional recovery included: experiencing a dysfunctional body, experiencing socio-cultural changes in relation to eating, and encountering organizational nutritional care delivery failures.
Chapple et al., 2017 [27] (Australia)	Prospective observational study	To describe changes in anthropometrics and nutritional status in TBI patients between ICU admission and 3 months post-admission.	Critically ill adults with TBI requiring ICU stay ≥ 48 h *n* = 37	**Body composition:** Day 7 post-ICU admission, weekly thereafter until hospital discharge (up to 3 months post-admission): Weight, ultrasound assessment of quadriceps muscle layer thickness **Global nutritional status assessment:** Day 7 post-ICU admission, weekly thereafter until hospital discharge up to 3 months post-admission: SGA **Functional status assessment:** SF-36v2	Between hospital admission and discharge, mean weight loss was ~ 5%; at 3 months following ICU admission, weight loss persisted. The slope of quadriceps muscle layer thickness loss was steepest during ICU admission, but largely recovered 3 months post-ICU admission. Proportion of patients moderately to severely malnourished at ICU admission: 14%; at hospital discharge 38%.
Chapple et al., 2018 [28] (Australia)	Qualitative approach; study not grounded in a specific qualitative methodological framework	To explore the views and attitudes of ICU and ward physicians and nursing practitioners regarding nutrition interventions for TBI patients.	Healthcare providers (18 nurses, 16 physicians) working in ICU or on ward with post-ICU patients *n* = 34	N/A	Barriers to nutrition interventions identified by nurses and physicians related to use of feeding tubes, competing priorities of care/nutrition not a top priority, lack of education regarding importance of nutrition.
Merriweather et al., 2018 [29] (Scotland)	Secondary analysis of the RECOVER RCT [24].	To investigate changes occurring in appetite over 12 weeks following ICU discharge.	Critically ill adults requiring > 48 h MV *n* = 193	**Factors affecting intake:** ICU discharge, 3-, 6-, and 12-month follow-ups: Appetite VAS	Appetite is suppressed (< 5 cm on VAS) at the time of ICU discharge, with no improvements seen throughout hospitalization. 3-months post-discharge, appetite scores increased by 1.7 cm but remained low.
Jarden et al., 2018 [30] (New Zealand)	Service evaluation	Assess oral intake in post-ICU patients up to 1 month following extubation.	Patients admitted to a mixed ICU requiring intubation *n* = 79	**Oral intake:** Meal intake (proportion of meal tray and use of ONS) assessed daily using intake audit records measured during hospitalization following ICU discharge	62% of patients had inadequate oral intake, defined as consuming < 2/3 of their meal tray, and of these patients, 60% consumed < 1/3 of their meals. One quarter of patients were unable to feed themselves independently.
Ridley et al., 2019 [31] (Australia)	Nested cohort study	Assess dietary intake and assess energy expenditure in the post-ICU hospitalization period.	Critically ill adults with ≥ 1 defined organ system failure requiring MV *n* = 32	**Energy and protein intake:** EN/PN: calculated daily from flow sheets in the medical record Oral diets: 24-h recall, food record chart review **Estimation of requirements to assess adequacy:** Indirect calorimetry (n = 12); 25–30 kcal/kg calculated body weight (*n* = 20) set as actual body weight if BMI < 25 kg/m^2^ or ideal body weight of BMI 23 kg/m^2^ if BMI ≥ 25 kg/m^2^	Patients receiving oral diets without ONS consumed median 37% and 48% of prescribed calories and protein, respectively. Patients receiving oral diets including ONS consumed median 73% and 68% of prescribed calories and protein, respectively. Adequacy of calorie and protein intake for patients on EN and EN with an oral diet was 62% and 59%, respectively, and 104% and 99%, respectively.
Chapple et al., 2019 [32] (Australia)	Inception cohort study	Evaluate dietary intake, appetite, and gastric emptying 3 months following ICU discharge.	Adults requiring an ICU admission and alive at hospital discharge *n* = 51 ICU survivors *n* = 25 healthy controls	**Energy and protein intake:** Day prior to gastric emptying testing: 24-h recall conducted by a dietitian Following consumption of carbohydrate drink for gastric emptying test: standard buffet meal (participants ate from a standardized selection of food ad libitum until full) **Body composition:** Weight: self-reported prior to ICU admission; measured at 3-month follow-up (gastric emptying testing day) **Factors affecting intake:** Appetite VAS Gastric emptying via carbohydrate absorption and breath testing	ICU survivors consumed fewer kcal the day prior to testing, as evaluated using the 24-h recall; on the testing day, intake, assessed via the weighed buffet, did not differ between the groups. No differences in appetite rating or the rate of gastric emptying between ICU survivors and healthy controls were observed.
Wittholz et al., 2020 [33] (Australia)	Prospective observational study	Assess nutritional outcomes in trauma patients following ICU discharge.	Critically ill adult trauma patients requiring MV > 48 h *n* = 28	**Energy and protein intake:** Determined daily from days 1–5 post-ICU discharge EN/PN: calculated from daily fluid balance charts Oral diets: visual estimation of meal components consumed **Estimation of requirements to assess adequacy:** Dietitian prescriptions **Body composition:** Measures taken at ICU discharge and weekly until day 26 or hospital discharge: Weight, QMLT via ultrasound **Global nutritional status assessment:** Hospital discharge: SGA **Functional status assessment:** Measure taken at ICU discharge and weekly until day 26 or hospital discharge: hand-grip strength	Patients receiving oral diets consumed a mean of 54% and 65% of prescribed calories and protein, respectively. Adequacy of calorie and protein intake for patients receiving artificial nutrition (EN, EN with an oral diet, PN) was 87% and 87%, respectively.
Moisey et al., 2021 [34] (Canada)	Prospective observational study	Assess nutritional intake up to 14 days following LMV.	Critically ill patients from a mixed ICU requiring MV > 72 h *n* = 19	**Energy and protein intake:** EN/PN: calculated daily from flow sheets in the medical record up to day 90 of hospitalization Oral diets: weighed food records for 7 days and day 14 following LMV **Estimation of requirements to assess adequacy:** Energy and protein prescriptions assessed by the hospital dietitians as part of standard care were extrapolated from the charts **Barriers to intake:** Identified using non-validated checklist of barriers to eating commonly identified in hospitalized patients	Patients receiving oral diets consumed median 47% and 27% of prescribed calories and protein, respectively. Adequacy of calorie and protein intake for patients on EN and EN with an oral diet was 100% and 100%, respectively, and 74% and 75%, respectively. Primary barriers to eating identified included poor appetite, early satiety, taste changes, nausea/vomiting, and disliked the food served.

*ALI* Acute lung injury, *BMI* body mass index, *EN* enteral nutrition, *GRA* generic rehabilitation assistant, *ICU* intensive care unit, *MAC* mid-arm circumference, *MAMC* mid-arm muscle circumference, *LMV* liberation from mechanical ventilation, *MV* mechanical ventilation, *NGT* nasogastric tube, *ONS* oral nutrition supplements, *PEG* percutaneous endoscopic gastrostomy, *PN* parenteral nutrition, *QMLT* quadriceps muscle layer thickness, *RCT* randomized control trial, *SGA* Subjective Global Assessment, *TBI* traumatic brain injury, *TSF* triceps skinfold, *VAS* visual analogue scale

### Biological barriers to nutrition intake during recovery from critical illness

ICU survivors experience a multitude of barriers to optimizing nutritional recovery due to several physiological and psychological factors.

#### Barriers to nutrition intake: physiological

The most reported barrier to eating post-extubation is poor appetite [19, 20, 22, 29, 32, 34], which can persist several months following ICU discharge [29, 32, 72]. Poor appetite is associated with reduced dietary intake, as well as increased comorbidities in recovery [72]. Other physiological factors affecting nutrition that have been consistently reported as issues of concern in the recovering critically ill include early satiety [19, 32, 34], nausea and vomiting [19, 20, 34], and taste changes [23, 26, 34].

#### Barriers to nutrition intake: functional

Another major barrier ICU survivors face is swallowing difficulties typically associated with oropharyngeal dysphagia (OPD) related to orotracheal intubation and ICU-acquired weakness [73–75]. Up to 84% of patients may be diagnosed with post-extubation OPD [74, 76], and a recent scoping review by Skoretz et al. [73] reported between 11 and 83% of patients with a tracheostomy will have OPD. OPD is associated with malnutrition [77, 78], prolonged hospital stays [79, 80], and increased mortality [79]. Dietary intervention for patients with OPD centers on the prescription of modified texture and fluid diets (for example, puréed food and thickened liquids) [81, 82] which are associated with reduced calorie and protein intake [81, 82] putting patients at higher risk of developing or worsening malnutrition. The time between extubation and initiation of any type of oral diet is longer in patients with OPD than those without [83], further compromising the nutritional status of ICU survivors in the early phases of recovery. Some patients experience severe OPD, prohibiting consumption of any solids or liquids due to aspiration risk; these patients are likely to have percutaneous feeding tubes inserted [81, 82].

ICU-acquired weakness stems from significant muscle wasting, decreased muscle contractile strength and neuropathies, the cause of which is directly related to critical illness itself [2, 84, 85]. It is associated with reduced ability or complete loss of volitional feeding [4, 26, 86] which is often coupled with reduced dietary intake [26, 87]. Inability to feed oneself independently persists well into the recovery phase of illness. Herridge and colleagues found in a cohort of ICU patients that approximately one-third of patients aged 44 and above who experienced an ICU LOS greater than two weeks were unable to feed themselves independently 6 months following ICU discharge [4].

#### Barriers to nutrition intake: psychological

Many recovering critically ill patients face substantial psychological disability or are diagnosed with mental health disorders including depression, anxiety, and post-traumatic stress disorder [13, 88–90]. In the early phases of ward-based recovery, it has been found that low mood and anxiety negatively impacted food intake predominantly because eating was viewed as low priority while patients struggled to cope with the drastic changes in their health [26]. Other factors contributing to poor nutritional intake or decreased appetite experienced by ICU survivors include body dysmorphia, sleep disturbances, pain, and fatigue [26, 72].

### Non-biological barriers to nutrition intake during recovery from critical illness

#### Organizational barriers

The ability of any hospitalized patient to consume adequate nutrition can be negatively affected by organizational barriers including delivery of meals at inappropriate times, missed meals and snacks, and interrupted mealtimes [91–93]. The lack of flexibility with hospital meal times has been identified as suboptimal for the critically ill patient who is suffering from altered sleeping patterns/disturbances, as well as a poor appetite coupled with early satiety [23, 26]. Care transitions out of the ICU and from hospital (Fig. 1) can also influence nutrition recovery.

#### Healthcare provider knowledge

Transfers out of the ICU often coincide with a transfer of care between health care providers and transfers to units where the staff-to-patient ratio is reduced and staff may lack specialized knowledge to provide the complex care required for an ICU survivor [94, 95]. Nutrition care plans are poorly communicated between health care providers, ward staff do not have sufficient knowledge of the specific nutrition needs of a critically ill patient, nutrition care is sometimes viewed as lower priority when compared with medical needs, and nasogastric feeding tubes are frequently removed prior to any dietetic assessment [23, 28]. Each of these factors significantly hinders the nutritional rehabilitation of survivors of critical illness.

### Nutrition intake during the recovery phase of critical illness

Given the multitude of barriers patients face to optimizing their nutritional health, it is not surprising that nutritional intake is often compromised in ICU survivors.

#### Calorie and protein intake during the post-ICU hospitalization period

**Fig. 3:**
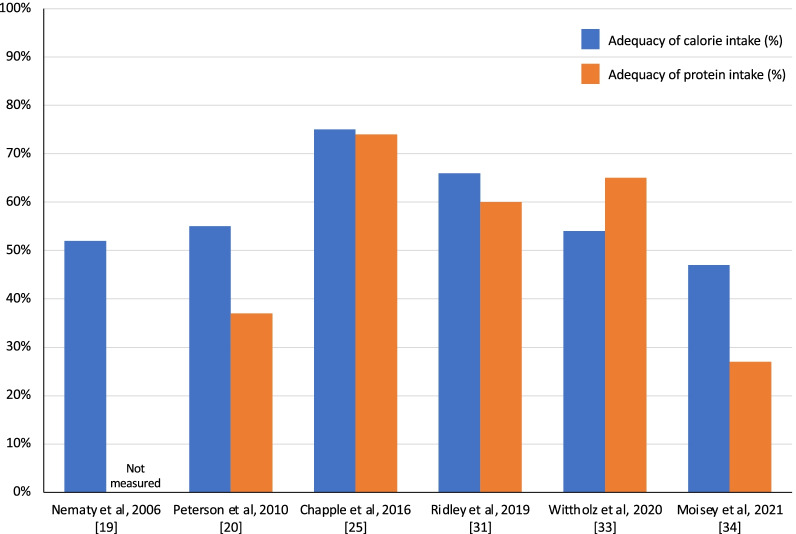
Adequacy of calorie and protein intake in relation to estimated or prescribed amounts in patients solely prescribed oral diets in hospital following ICU discharge

Measures of calorie and/or protein intake in various critically ill patient populations post-extubation and after ICU discharge have been quantified and reported in eight studies [19, 20, 22, 25, 30, 31, 33, 34] (Table 1) which focused on intake in the post-ICU hospitalization period. Enteral nutrition is the preferred route of feeding in/for critically ill mechanically ventilated patients [96, 97]; however, feeding tubes are often removed at the time of extubation or shortly thereafter, leaving patients solely reliant on oral intake to meet their nutritional needs. Studies have consistently demonstrated that patients relying on an oral diet alone during the early phases of ward-based recovery consume between 55 and 75% of prescribed calories and 27–74% of prescribed protein [19, 20, 25, 31, 33, 34] (Fig. 3), further contributing to the calorie and protein deficits that are commonly accrued in ICU [25, 98, 99]. In contrast, patients who continue to receive enteral nutrition, with or without an oral diet, fare considerably better, receiving 62–104% of prescribed calories and 59–100% of prescribed protein [22, 25, 31, 33, 34]. Consequently, removal of feeding tubes at or near the time of extubation should be avoided.

#### Calorie and protein intake following hospital discharge

To date, two studies have reported on calorie and protein intake at a single timepoint following ICU discharge [22, 32]. In a pilot feasibility study by Salisbury et al. [22] examining whether the use of a generic rehabilitation assistant could enhance physical and nutrition rehabilitation, dietary intake was evaluated in both the control (usual care) and intervention (use of a generic rehabilitation assistant) groups 3 months post-hospital discharge. They found that patients in the control and intervention groups were consuming a median 70% and 113% of estimated calorie requirements, respectively, and 69% and 90% of estimated protein requirements. In the second study, Chapple et al. [32] compared differences in dietary intake, appetite, and gastric emptying between ICU survivors at three months post-ICU discharge and a group of healthy controls that did not differ statistically for age or weight. They found usual dietary intake, measured via a 24-h recall, to be significantly lower in the ICU survivors versus the healthy controls; however, they did not express intake in relation to participants’ estimated energy and protein requirements. Currently, no longitudinal studies with repeated measurements over time exist in which nutrition intake (e.g., daily caloric, macronutrient or micronutrient intake) has been quantified in ICU survivors following discharge from hospital and movement into the longer-term phases of recovery, highlighting a considerable knowledge gap in the literature.

## Pragmatic strategies to enhance nutrition recovery and rehabilitation in ICU survivors

Despite the diversity between the studies reviewed with respect to geographical origin and methodologies to assess indices of nutritional status (Table 1), there is a remarkable consistency in their findings pertaining to the challenges of optimizing nutrition recovery and rehabilitation in post-ICU patients. As such, strategies to enhance nutrition recovery and rehabilitation that may be widely applicable to various settings are discussed below.

### Patient-centered nutrition care and family engagement

Provision of nutritional care on the ward traditionally takes a service centered approach where care is organized around the service and not the patient. To better meet patient needs, a more patient-centered approach should be adopted. Manley et al. [100] highlighted that a patient-centered approach should incorporate: seeing a patient as a person and learning of their needs, values, beliefs; promoting patient autonomy by enabling the patient to make informed decisions, including adapting and tailoring information to the patient to assist in the decision making process; shared decision making should occur between the healthcare team and the patient; advocating for the patient; and care provided to the patient is continually evaluated and feedback from patients is acted upon. To facilitate patient-centered processes, consideration must also be given to the care environment [101]. For example, post-ICU patients often have problems in sleeping; therefore, provision should be made for a later breakfast. Visiting hours should be adapted to include mealtimes so that family members an aid with feeding and relatives can play an important role in the social facilitation of eating. Tables and chairs should be provided to allow patients to eat together rather than in or beside their bed. As part of this approach, the patient and family will require education about nutritional needs after critical illness with information and feedback given around setting and achieving nutritional targets. Marshall and colleagues have demonstrated that engaging families in nutrition care by providing nutrition education, having them to complete food diaries, and advocate for nutrition care, is a feasible and acceptable approach to optimize nutrition intake during recovery from critical illness [102, 103]

### Healthcare provider education

The complexity of the nutritional problems faced by patients after critical illness is currently not recognized or addressed by ward staff [23, 28]. Standard nutritional care provision on a ward is typically focused on the general hospital patient population and may not meet the needs of post-ICU patients. The effects of critical illness impacts on the nutritional intake of patients through several interrelated issues with the body, sociocultural aspects of eating, and the organization of care. Education should focus on the issues that may be experienced by post-ICU patients especially during the early phase of their ward stay such as poor appetite, early satiety, taste changes, weakness, and fatigue, and offer practical suggestions to circumvent these problems. Details would also be provided about the common psychological problems experienced after critical illness and how these may affect nutritional intake. Provision of optimal nutrition care of ICU survivors must be addressed in both the graduate and post-graduate education of healthcare providers including nurses, physicians, and dietitians.

### Improving care transitions

#### Transitioning from the ICU to the ward

A clearly documented nutritional management plan should be handed over to ward staff and appropriate allied health professionals. This nutritional management plan should include any issues influencing nutritional intake in ICU including physiological factors such as poor appetite, early satiety, taste changes, weakness, fatigue, or psychological issues and delirium. Other information on the management plan should include a description of the patient’s current nutritional intake incorporating nutrition from parenteral, enteral, and oral routes, food likes and dislikes, and details about family involvement in nutritional care. Specific recommendations should be clearly communicated to reduce ill-informed decision making by ward-based staff. These recommendations may include the need to continue with enteral feeding or nutritional supplements.

#### Transitioning from hospital to home or other discharge destination

With a complex patient population, there is a clear need for well-coordinated discharge planning including the provision of tailored dietary advice such as information about caloric and nutrient dense foods and oral nutritional supplements. Ongoing nutritional care needs should be transferred to community dietetic services. Critical care recovery services and post-ICU clinics exist to provide outpatient follow-up to ICU survivors. These clinics are typically tailored to address the ongoing health issues of ICU survivors and their families. While they are multidisciplinary in nature, the composition of health disciplines represented varies. Given the ongoing nutrition concerns many ICU survivors face, it is important that dietitians be included in any post-ICU care delivery models.

## Looking forward: research opportunities in post-ICU nutrition rehabilitation

The study of nutrition in ICU survivorship remains in its infancy with much work to be done. Longitudinal studies that examine various facets of nutrition in ICU survivors spanning from ICU to the months and years following hospital discharge are needed. Such facets include:

- Examination of survivors’ intake and assimilation of calories and nutrients, and elucidating their nutritional requirements (e.g., protein and calories) over the recovery trajectory.
- Further characterization of barriers and facilitators to optimizing a patient’s nutrition status in recovery.
- Characterizing long-term changes in nutritional status, including body composition.
- Evaluation of the delivery of nutrition care and services.
- Garnering a more comprehensive understanding of patient and family perspectives relating to their perceptions of nutrition in ICU recovery.

Recognition that nutrition care is an important component of rehabilitation is underappreciated in ICU survivors despite the interplay between nutrition, physical and psychological health, and quality of life. Very few studies testing rehabilitation strategies to mitigate the impact of PICS and improve physical and psychological health outcomes in ICU survivors have incorporated a nutrition component within the rehabilitative strategy. Failure to assess and optimize the nutritional status of those who are enrolled in a physical rehabilitation program may limit the efficacy of the intervention. Research is needed to develop novel nutritional interventions that consider the multitude of factors that have been highlighted in this review in order to maximize the likely impact of the intervention. Potential nutritional and nutraceutical interventions may include agents that promote muscle protein synthesis or a shift toward an anabolic environment (e.g., branched chain amino acids, β-hydroxy-β-methylbutyrate, and creatine), exhibit anti-inflammatory properties, regulate appetite and satiety, or promote neuronal synaptic plasticity along the gut–brain axis. Development and testing of combined nutrition and physical rehabilitation or exercise interventions are warranted in the recovering critically ill as such models have been successful in increasing muscle mass and strength in comparison with nutrition or exercise-only interventions in other clinical populations [104, 105].

## Conclusions

The role of nutrition in the recovery phase of critical illness has received increasing attention over the last few years; however, it remains underrecognized and underappreciated. Studies have consistently demonstrated that nutritional intake is suboptimal in ICU survivors with a myriad of factors influencing nutritional recovery. Future work should include novel interventions to address these barriers to facilitate nutritional rehabilitation.

## Data Availability

Not applicable.

## Abbreviations

ICU: Intensive care unit
OPD: Oropharyngeal dysphagia
PICS: Post-intensive care syndrome
